# Genome, transcriptome and proteome: the rise of omics data and their integration in biomedical sciences

**DOI:** 10.1093/bib/bbw114

**Published:** 2016-11-22

**Authors:** Claudia Manzoni, Demis A Kia, Jana Vandrovcova, John Hardy, Nicholas W Wood, Patrick A Lewis, Raffaele Ferrari

**Affiliations:** 1 School of Pharmacy, University of Reading, Whiteknights, Reading, United Kingdom; 2 Department Molecular Neuroscience, UCL Institute of Neurology, London, United Kingdom

**Keywords:** omics, bioinformatics, databases, genomics, transcriptomics, proteomics

## Abstract

Advances in the technologies and informatics used to generate and process large biological data sets (omics data) are promoting a critical shift in the study of biomedical sciences. While genomics, transcriptomics and proteinomics, coupled with bioinformatics and biostatistics, are gaining momentum, they are still, for the most part, assessed individually with distinct approaches generating monothematic rather than integrated knowledge. As other areas of biomedical sciences, including metabolomics, epigenomics and pharmacogenomics, are moving towards the omics scale, we are witnessing the rise of inter-disciplinary data integration strategies to support a better understanding of biological systems and eventually the development of successful precision medicine. This review cuts across the boundaries between genomics, transcriptomics and proteomics, summarizing how omics data are generated, analysed and shared, and provides an overview of the current strengths and weaknesses of this global approach. This work intends to target students and researchers seeking knowledge outside of their field of expertise and fosters a leap from the reductionist to the global-integrative analytical approach in research.

## Introduction

**Figure 1.**
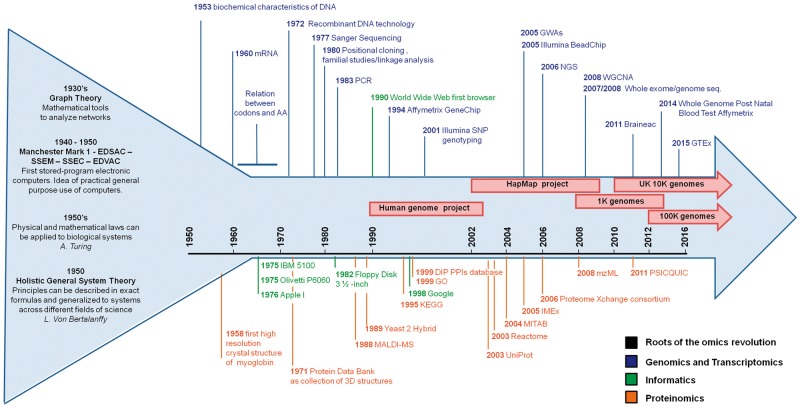
Overview of the progressive advance in the methods to study genes, transcripts and proteins in the informatics sciences. The arrow represents the development, over time, of the many disciplines now involved in biomedical science accompanied by the fundamental advances in informatics and community resources. The broad roots of the omics revolution are represented by the wider start of the arrow before the year ‘1950’, when the foundations for a paradigm shift in science (from single observations to systems dynamics) were laid.

The exponential advances in the technologies and informatics tools (Figure 1) for generating and processing large biological data sets (omics data) is promoting a paradigm shift in the way we approach biomedical problems [1–10]. The opportunities provided by investigating health and disease at the omics scale come with the need for implementing a novel *modus operandi* to address data generation, analysis and sharing. It is critical to recognize that (multi)omics data, that is, omics data generated within isolated and not yet integrated contexts, need to be analysed and interpreted as a whole through effective and integrative pipelines [integrated (multi)omics, then referred to as integromics or panomics [11]]. This clearly requires the cooperation of multidisciplinary teams as well as the fundamental support of bioinformatics and biostatistics. Nevertheless, in the midst of such change in study approach, we currently experience the establishment of fragmented niche groups who each developed specific jargons and tools, a fact that inevitably impacts the flow of information and the communication between different teams of experts (e.g. physicians, researchers, bioinformaticians and biostatisticians), and, eventually, data interpretation.

In this scenario, our review intends to be a cross-disciplinary survey of omics approaches with a particular emphasis on genomics, transcriptomics and proteinomics. We provide an overview of the current technologies in place to generate, analyse, use and share omics data, and highlight their associated strengths and pitfalls using an accessible language along with illustrative figures and tables. We have summarized critical considerations in Tables 1 (general), **2** (genome), **3** (transcriptome), **4** (proteome) and **5** (functional annotations); nevertheless, the readership shall keep in mind that these reflect authors’ views and are not intended to be exhaustive. Useful web-based resources are included in Supplementary Tables S1a–e, and a comprehensive **Glossary** is provided in the Supplementary Files. All this allows reaching a broad audience, including researchers, clinicians and students, who are seeking a comprehensive picture of research-associated resources beyond their background or speciality. In summary, we here intend to stress a conscious way of thinking in the view of the rise of data integration from multidisciplinary fields, a fact that is fostering a leap from the reductionist to the global-integrative approach in research.

**Table 1.** General critical considerations on applying bioinformatics to the biomedical sciences. Problems that can be addressed by individual researchers or research groups or that should be addressed by a large community effort have been flagged with * or °, respectively.

**Observation**	**Problems**	**Proposed action**
*Online tools are used with little to no criticism	Using inappropriate tools for a particular analysis Using default settings that may not be tailored for the research purpose Accepting an output without much criticism, leading to mis/over-interpretation of results	For informaticians: make the description of the tool as simple as possible For end user: understand the principles underlying a tool before using it
*Analysis can be run with different, though equally valid, algorithms and statistical methods	The wealth of tools available feeds the temptation to pick the one that either has the friendliest user interface or gives the most interesting result Results obtained using different tools are different	As with technical replicates in a wet laboratory, a good bioinformatics analysis must give consistent results even with different methods Repetition of the analysis with different tools supports consistency and reproducibility of findings
*Analysis may require the subjective selection of *a priori* parameters [12]	Same tools used by implementing different parameters will likely generate different results	Perform sensitivity analysis using alternative parameters
°Databases are on-going projects	Databases are constantly updated Analytical tools that rely on databases may become out of date if their libraries are not updated periodically Published bioinformatics analyses become out of date because of advances in the databases/reference sets	Use software and online tools with recent/frequent updates Bioinformatics analyses are complete only to the extent of the completeness of the reference database used Always document the software version and codes used for a particular analysis Code maintainers should keep archival copies of old software and code versions (if replications are necessary)
*Statistical methods were originally designed for ‘small’ scale data	If statistical methods are tailored for small-scale data, eventually the *p*-value will reach the pre-defined significance level in large-scale data sets risking spurious results	Be cautious in the statistical approaches used, and ask guidance from experts
°Analytical tools	Some of the resources are accessible only after a fee is payed. This very much limits their use to exclusively niche or research groups with funds for bioinformatics analysis	Free omics data access and usage is fundamental for reducing the fragmentation of research and stimulating the improvement of data integration, analysis and interpretation Foster open data policies with the support of governments and funding agencies
*Hypothesis-driven analyses	Results might be biased based on initial hypothesis Some outcomes might be inflated because of excessive targeting through the research tools being used (primers or probes, particular protein interactions, tissue-specific data)	Consider whether the experiment or analysis needs to be hypothesis driven or can be hypothesis free; use the right techniques/tools and analysis to address the research question (microarray versus NGS, association versus rare variants analysis, tissue-specific versus all tissues, eQTL versus epigenomics, etc)
*Experimental design	Wrong experimental design (without considering power calculation, adequate controls, tissue types, single cells versus tissue homogenates, etc) may lead to biased or underpowered results	Like any experiment, the analysis should be planned within a properly developed pipeline that takes into account data source, sample size, controls, techniques to generate data, analyses to apply to data

## The genome and genomics

Genomics is the study of organisms’ whole genomes (WGS). In *Homo sapiens*, the haploid genome consists of 3 billion DNA base pairs, encoding approximately 20 000 genes. These make up the coding regions (1–2% of the entire genome), while the remaining 98–99% (non-coding regions) holds structural and functional relevance [4, 13, 14].

### Genome features and genetic variants

**Figure 2.**
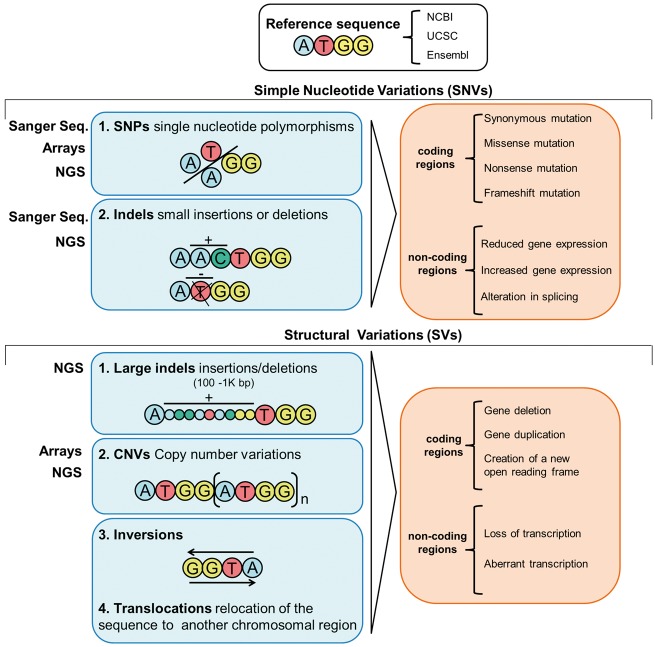
Overview of the types of variants in the genome, their potential consequences and the methods/techniques to untangle them.

Many factors influence the state of health and disease, and yet, it is clear that an individual’s genetic background is an important determinant. Examining this genetic background is, therefore, of great importance for identifying individual mutations and/or variants underpinning pathways that discriminate health and disease [4]. Since the elucidation of the structure of DNA [10], genetic and, latterly, genomic data have been generated with increasing speed and efficiency, allowing the transition from studies focused on individual genes to comparing genomes of whole populations (Figure 1) [15]. Many variants exist in the genome, the majority of which are benign; some are protective, conferring an advantage against certain conditions [16]. However, others can be harmful, increasing susceptibility for a condition (i.e. a cluster of variants with low penetrance) or directly causing a disease (i.e. one or few variants with high penetrance) [17]. The variants can be broadly categorized into two groups: simple nucleotide variations (SNVs) and structural variations (SVs). The former comprises single nucleotide variations and small insertion/deletions (indels); the latter includes large indels, copy number variants (CNVs) and inversions [18]. SNVs and SVs found in coding regions may impact protein sequence, while those in non-coding regions likely affect gene expression and splicing processes (Figure 2) [19]. These variants are often grouped into rare (frequency in the general population < 1%) and common (frequency > 1%); common single nucleotide variations are often referred to as single nucleotide polymorphisms (SNPs). Coding and non-coding portions as well as types of variants present within the genome have undergone an attentive nomenclature standardization to allow harmonized scientific communication. Working groups such as the Human Genome Organization gene nomenclature committee [20] or the Vertebrate and Genome Annotation projects [21] provide curation and updates on the nomenclature and symbols of coding and non-coding loci, whereas the standardized reference to properly code genetic variations is curated by the Human Genome Variation Society [22].

### Technologies and methods for genetics analysis

Current techniques to capture genetic variants such as SNVs and SVs include (i) Sanger sequencing [23], the base-by-base sequencing of a locus of interest that captures up to 1 kb per run; (ii) DNA-microarrays, based on hybridization of the DNA sample with a set of pre-defined oligonucleotide probes distributed across the entire genome or enriched around regions of interest [24]; and (iii) next-generation sequencing (NGS) methods based on the fragmentation of the genomic DNA into pieces that are subsequently sequenced and aligned to a reference sequence [25]. Microarrays are less costly than NGS, yet both strategies allow the identification of SNVs as well as some types of CNVs (Figure 2); nevertheless, microarrays are more limited comparatively to NGS strategies, as they are based on *a priori* knowledge of sequence and SNVs, while NGS allows detection of novel changes. Particularly, NGS allows the sequencing of specifically targeted regions, whole exome (WES) and WGS of individuals. WES allows the screening of all variants (including rare) in the coding region with a direct relation to protein affecting mutations; WGS allows the identification of all rare coding and non-coding variants [19, 25].

The study of the genome relies on the availability of a reference sequence and the knowledge of the distribution of the common variants across the genome. This is important to (i) map newly generated sequences to a reference sequence and (ii) refer to population-specific genetic architecture for interpretation of studies such as genome-wide association studies (GWAS) [26]. The human genome was sequenced through two independent projects and released in the early 2000s by the public Human Genome Project (HGP) and a private endeavour led by J. Craig Venter; as a result, the human reference sequence was constructed and over 3 millions SNPs were identified [4, 14]. The Genome Reference Consortium, a successor of the HGP, maintains and updates the reference genome, which is currently in build GRCh38 [also referred to by its University of California Santa Cruz (UCSC) Genome Browser version name hg38] [27, 28]. The reference genome is paired with a genome-wide map of common variability, thanks to the International HapMap Project (Figure 1) [3]. This project identified common variants [minor allele frequency (MAF) ≥ 5%] across the genome of different populations (African, Asian and European ancestry), leading to the awareness that up to 99.5% of the genome across any two individuals is identical and to the mapping of up to 10 millions SNPs. Importantly, the HapMap project allowed to complement the HGP with additional information such as that of haplotype blocks, based on the concept of linkage disequilibrium (LD, see glossary), the grounding foundation of GWAS [15]. To increase the resolution achieved by HapMap, the 1000 Genomes Project was concluded in 2015, with 2504 genomes sequenced from 26 populations [29] to produce an extensive public catalogue of human genetic variation, including rarer SNPs (MAF ≥ 1%) and SVs. This data (reference genome + HapMap + 1000 Genomes projects) are publicly available, greatly fostering high resolution and population-specific GWAS and filtering of benign common and rare variants for NGS data analysis. More recent projects such as UK10K [30], 100 000 Genomes Project [31] and the Precision Medicine Initiative [32] will further help to enhance our understanding of human genetic variability by identifying and annotating low-frequency and rare genetic changes.

A typical GWAS design involves using a microarray to genotype a cohort of interest and to identify variants associating with a particular trait in a hypothesis-free discovery study. A GWAS results in a list of SNPs evaluated for their frequency in relation to the trait under study; most reported associations in GWAS are intronic or intergenic, affecting DNA structure and gene expression rather than protein sequence [17]. GWAS identify risk loci, but not necessarily the prime variants or genes responsible for a given association (due to LD), nor their function. Replication and targeted re-sequencing approaches are required to better understand the association found in the discovery phase. Nevertheless, a GWAS suggests potential biological processes (BPs) associated with a trait to be further investigated in functional work [26].

The explosive growth in the number of GWAS in the past 10 years has led to the discovery of thousands of published associations for a range of traits (25 342 unique SNP-trait associations from 2586 studies in GWAS catalogue as of October 2016). These studies have both confirmed previous genetic knowledge (e.g. for Parkinson’s disease α-synuclein and Leucine Rich Repeat Kinase 2 were firstly identified to be segregating in affected families, and then replicated in GWAS [33]) and suggested novel loci. Although most of the associating SNPs have a small effect size, they provide important clues on disease biology and even may suggest new treatment approaches (e.g. in sickle-cell disease, *BCL11A* was identified as a gene controlling foetal haemoglobin levels [34, 35]; in Crohn’s disease, GWAS underlined the pathogenic role of specific processes such as autophagy and the innate immunity [36]). Another opportunity supported by GWAS is the possibility of comparing the genetic architecture between traits (LD score regression [37]). Conversely, a common criticism is that significant SNPs still do not explain the entire genetic contribution to the trait (i.e. missing heritability [38]); however, models incorporating all SNPs regardless of their statistical significance in GWAS, substantially improve the genetic explanation of the trait [39] for which, ultimately, the remaining missing heritability is likely explained by rare variants (therefore not captured in GWAS).

Traditionally, GWAS has been performed through microarrays, and, although NGS methods are becoming increasingly popular due to a reduction in the cost of the technology, the economical impact of WES and WGS is still around 1–2 orders of magnitude more than that of a genome-wide microarray, making the latter still preferable, particularly, for the genotyping of bigger cohorts. However, a valuable option that is gaining momentum is that of combining the two techniques: NGS is, in fact, extremely helpful together with genotyping data (within the same population) to increase the resolution of population-specific haplotypes and strength of imputation [40]. In summary, the choice between a microarray or NGS approach should be based on the scientific or medical question(s) under consideration, for which pertinent concepts can be found in [26, 41, 42].

### Tools for genomics analysis

Many tools are available for handling genome-wide variant data (e.g. Plink [43], Snptest [44] and a variety of R packages, including the Bioconductor project [45]) supporting the whole workflow from quality control (QC) of raw genotyping data to analysis, such as association, heritability, genetic risk scoring and burden analyses. In particular, Bioconductor is a valuable resource for using and sharing packages and/or pipelines. NGS data undergo different QC steps with dedicated programs such as the Genome Analysis Toolkit to align the sequences with the reference genome, and to call and filter rare variants [46]. Valuable resources are available to catalogue both GWAS and NGS data. For example, the GWAS catalogue, curated by the National Human Genome Research Institute, European Molecular Biology Laboratory-European Bioinformatics Institute (EMBL-EBI) and National Center for Biotechnology Information (NCBI; based in the United States), is freely available on the EBI website for exploring genetic associations within a variety of traits. Of note, a comprehensive repository of all currently available genetic variations (including links to the original studies) is curated by EBI within the European Variation Archive [47]. The Exome Aggregation Consortium is an online tool for evaluating WES results (i.e. exploring genes, variant types and frequencies and predicted effects). Other useful repositories are the NCBI, Ensembl and UCSC portals: each comprises resources to explore genes, variants and their associated effects. For example, dbSNP (within NCBI) provides comprehensive information about SNPs including location, effect and population-specific prevalence. ClinVar or Online Mendelian Inheritance in Man (also within NCBI) helps in associating coding variants with traits and provides a comprehensive review on links between genetic variability and diseases, respectively. Biomart (within Ensembl) allows for filtering and extracting information of interest for a particular gene or SNP. Furthermore, these repositories provide the opportunity to link and display genetic and transcript data together, e.g. on Ensembl or UCSC. Other repositories include dbGap (in the United States; NCBI) or the European Genome-Phenome Archive (in Europe; EMBL-EBI) where data from individual studies can be submitted. Policies to regulate data access and usage might apply (see ‘Perspectives’ section for further details), and vary from institution to institution. In some cases, data are only available by contacting groups or consortia generating data.

We have summarized critical considerations in Table 2, and all web resources included in this section are shown in Supplementary Table S1a.

**Table 2.** General critical considerations on applying bioinformatics to genomics. Problems that can be addressed by individual researchers or research groups or that should be addressed by a large community effort have been flagged with * or °, respectively.

**Observation**	**Problems**	**Proposed action**
*Genome build	Analysing data using inconsistent genome builds can lead to spurious results	Use the correct genome build when mapping
*Multiple databases [48]	Difficult to select among the many databases that exist	Investigate their limitations, including lack of corrections or updates to annotations
°Large-scale databases [49]	With large-scale data there may be a decrease of phenotype data quality	Consider case ascertainment methods and length of follow-up of controls
°Variant effect prediction	Effect prediction tools are not infallible	Verify segregation, absence from controls and *in vitro* effect of coding variants
°Linear genome reference [50]	A linear genome reference is not representative across individuals and populations	GRCh38 addresses this issue by providing alternative sequence representations for regions where a consensus sequence is difficult to be determined

## The transcriptome and transcriptomics

The transcriptome is the total complement of ribonucleic acid (RNA) transcripts in a cell and consists of coding (1–4%—messenger) and non-coding (>95%—ribosomal, transfer, small nuclear, small interfering, micro and long-non-coding) RNAs [51, 52]. Provided the tailoring of *ad hoc* techniques and the growth of recent data on coding RNAs (mRNAs), these will be the main focus of this section.

### Technologies and methods for transcriptomics analysis

The analysis of mRNAs provides direct insight into cell- and tissue-specific gene expression features such as (i) presence/absence and quantification of a transcript, (ii) evaluation of alternative/differential splicing to assess or predict protein isoforms and (iii) quantitative assessment of genotype influence on gene expression using expression quantitative trait loci analyses (eQTL) or allele-specific expression (ASE). This information is fundamental for a better understanding of the dynamics of cellular and tissue metabolism, and to appreciate whether and how changes in the transcriptome profiles affect health and disease.

**Figure 3.**
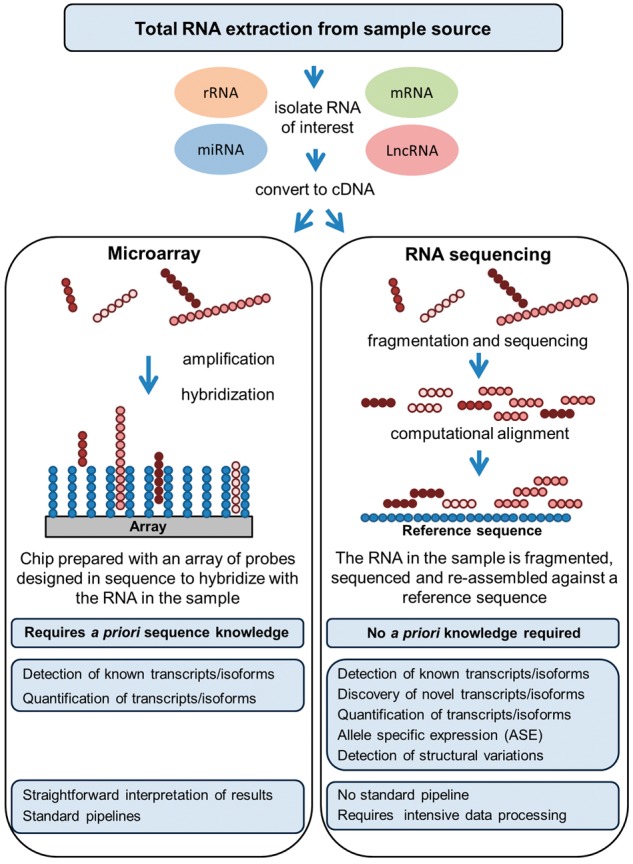
Summary of various features associated with either RNA-microarrays or RNA-sequencing data generation and analysis.

It is now possible to capture almost the totality of the transcriptome through similar strategies used for screening the DNA, i.e. microarray and sequencing techniques (Figure 3). As mentioned in the previous section, the RNA-microarray approach is less costly than RNA-sequencing but has significant limitations, as the former is based on previously ascertained knowledge of the genome, while the latter allows broad discovery studies [53]. RNA-microarrays are robust and optimized for comprehensive coverage through ever updated pre-designed probes; however, transcripts not included in the probe set will not be detected. Of note, although complementary accessories among the microarrays options, such as the tiling array, allow to characterize regions which are contiguous to known ones supporting the discovery of *de novo* transcripts [54], RNA-sequencing is more comprehensive, as it enables capturing basically any form of RNA at a much higher coverage [55].

The workflow to generate raw transcriptome data, through either method, involves the following: (i) purifying high-quality RNA of interest; (ii) converting the RNA to complementary DNA (cDNA); (iii) chemically labelling and hybridizing the cDNA to probes on chip (RNA-microarray) or fragmenting the cDNA and building a library to sequence by synthesis (RNA-sequencing); (iv) running the microarray or sequence through the platform of choice; and (v) performing *ad hoc* QC [55, 56].

The QC steps differ between microarray and sequencing data [56]: for the former, chips are scanned to quantify signals of probes representing individual transcripts, and reads are subsequently normalized; for the latter, the raw sequences are processed using applications such as FastQC that read raw sequence data and perform a set of quality checks to assess the overall quality of a run. This step is then followed by alignment with a reference sequence (to evaluate coverage and distribution of reads), transcript assembly and normalization of expression levels [57]. Different types of *post hoc* analyses can be performed with both microarray and sequencing data including differential cell- and/or tissue-specific expression profiles that test whether genes have different expression levels across tissues and conditions [56] or analyses investigating gene expression regulations such as eQTL analyses. eQTL mapping is a powerful approach to investigate and integrate gene expression measures (RNA-level data) with genetic variants (DNA-level data). Analysing eQTL helps to identify genetic variants that influence mRNA expression levels. As discussed in the previous section, GWAS hits (i.e. associated SNPs) are mainly located in non-coding regions and are thus likely to exert their function through the regulation of gene expression. The knowledge of eQTLs for GWAS loci can help in prioritizing causal variants, which are generally hard to fine map due to LD issues. It follows that eQTLs provide an important link between genetic variants and gene expression, and can thus be used to explore and better define the underlying molecular networks associated with a particular trait [58]. Most eQTLs identified to date regulate expression of a transcript or a gene in an allele-specific manner (*cis*-eQTLs): this regulation is local and often investigated within a ±1 mb upstream and downstream flanking regions of genes, limiting the number of statistical tests that need to be performed. In comparison, *trans*-eQTLs affect genes located anywhere in the genome and have weaker effect sizes: both features make *trans*-eQTL analyses currently difficult. During the past decade, the number of studies focusing on eQTL has exponentially grown and eQTL maps in human tissues have been and are being generated through large-scale projects [59–62]. Studying eQTLs in the right context is particularly important as eQTLs are often only detected under specific physiological conditions and in selected cell types. In this view, the development of induced pluripotent stem cells models is likely to advance our detection of physiologically and cell type-specific relevant eQTLs that are difficult to obtain form living individuals. In addition, it is important to note that a limitation of eQTL analysis, i.e. the need for a large number of samples to gain sufficient statistical power, can be overcome by ASE studies in which different expression levels between the maternal and paternal allele can be used for the investigation of effects of rare variants (e.g. protein affecting mutations) [63]. Of note, RNA-sequencing alone provides a framework for unique analyses investigating novel transcript isoforms (isoform discovery), ASE and gene fusions analyses [56]. Another way to study the regulation of gene expression is achieved through the combined analysis of mRNA and microRNA levels. MicroRNAs are short, non-coding RNA molecules that regulate the actual transcription of mRNA whose profiling is also captured both through array and sequencing techniques. The specific binding of microRNA to a target mRNA (by means of sequence homology) either inhibits mRNA binding to the ribosome or targets the mRNA for degradation. It is therefore clear that not only mRNA levels, but also their regulation by microRNAs are important for a more comprehensive overview on gene expression dynamics [64]. It is relevant to note that the specific microRNA content of a specimen might, *per se*, be predictive of a certain condition or trait and can therefore be immediately used in clinical diagnostics. However, microRNA profiling can be integrated with mRNA expression data to study changes in the transcriptome profile, specifically identifying the mRNA transcripts that undergo regulation, therefore highlighting the potential molecular pathways underpinning a certain trait or condition. One problem here, however, is the need to identify the mRNA molecules regulated by each given microRNA sequence for accurate visualization of gene regulatory networks [65]. There are tools, such as MiRNA And Genes Integrated Analysis web tool (MAGIA) [66], GenMiR ++ [67] and mirConnX [68], that are specifically developed to aid in this context. The mRNA/microRNA profiling approach has been, for example, successfully applied to study gene expression in specific subtypes of gastrointestinal tumours [69] or evaluate alteration of gene expression in wound-healing impaired fibroblasts from diabetic patients [70].

A more system-wide approach to assess gene expression is gained through gene co-expression analyses, including weighted gene co-expression network analysis (WGCNA) [71]. WGCNA assesses similarities in expression patterns, usually through Pearson’s or Spearman’s correlation, with the assumption that genes with similar expression profiles undergo similar regulation and are likely to share common biochemical pathways/cellular functions.

### Tools for transcriptomics analysis

There is a plethora of solutions for data storage, sharing and analysis. Groups that generate data store it either on private servers or public repositories. Examples of widely used portals to access and download data are, for example, the Gene Expression Omnibus, ArrayExpress or the Expression Atlas in EBI or the Eukaryotic Genome database in Ensembl. Such repositories, however, mainly allow for data storage and download (ethic requirements and policies might apply; see ‘Perspectives’ section for further details). Thus, the end user who downloads data needs to possess, or develop, a pipeline for analysis: Bioconductor is (again) a valuable resource for this. Other sites provide a framework for analysing data in an interactive and multi-layered fashion, such as NCBI, Ensembl and UCSC, or the Human Brain Atlas that allows verifying brain-specific expression patterns of genes of interest at different stages of life. The Genotype-Tissue Expression portal is a catalogue of human gene expression, eQTL, sQTL (splicing quantitative trait loci) and ASE data that can be used interactively to verify gene expression and gene expression regulation patterns in a variety of different tissues [59], while Braineac is a similar resource tailored for similar studies in human brain [61]. Other online tools are being developed and listed in *ad hoc* online portals, such as OMICtools, promoting the study of gene co-expression, translation initiation, interaction between RNA and RNA binding proteins (RBP), RNA editing, eQTL, *cis*- and *trans*-expression regulatory elements and alternative splicing to name a few.

We have summarized critical considerations in Table 3, and all web resources included in this section are shown in Supplementary Table S1b.

**Table 3.** General critical considerations on applying bioinformatics to transcriptomics. Problems that can be addressed by individual researchers or research groups or that should be addressed by a large community effort have been flagged with * or °, respectively.

**Observation**	**Problems**	**Proposed action**
°The transcriptome is cell-specific [72]	Use of RNA data from cells/tissues not specific for the aims of a study may lead to misleading results Many RNA data sets are based on tissue homogenates	Use RNA data obtained from source material relevant to the planned study Be aware of the possibility of contamination from different cell types in data originating from homogenates Establish a worldwide project for a bank of well-defined human cell lines representing all tissues and define their transcriptome at different times of the cell cycle to generate a ‘reference-transcriptome’
°The transcriptome is dynamic [73, 74]	The generalization of RNA data can lead to misleading interpretations	Be aware that data might reflect a particular cellular phase, or metabolism influenced by micro-environmental stimuli
°e/sQTLs depend on temporospatial variables [73, 74]	The generalization of e/sQTL results can lead to misleading interpretations	e/sQTLs depend on temporal (cell cycle/age) and spatial (cells/tissue/micro-environment) variables: consider these as covariates during data analysis and/or interpretation

## The proteome and proteinomics

**Figure 4.**
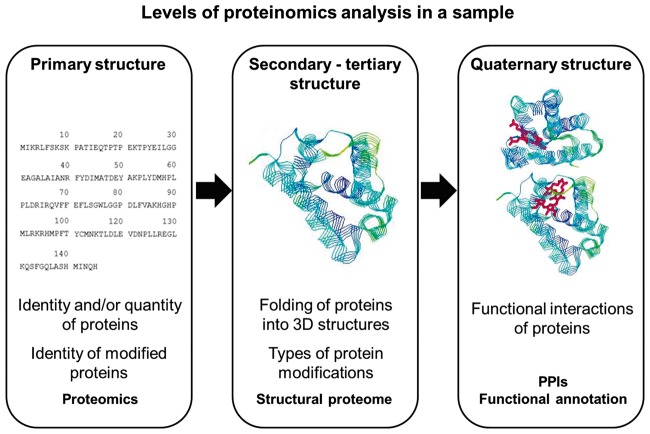
Summary of protein structural features and methods to generate and analyse proteomics data. The crystal structure of the haeme cavity of the haemoglobin of *Pseudoalteromonas haloplanktis* (4UUR [75]) was downloaded from PDB and visualized by RasMol (http://www.openrasmol.org/Copyright.html#Copying).

The proteome is the entire set of proteins in a given cell, tissue or biological sample, at a precise developmental or cellular phase. Proteinomics is the study of the proteome through a combination of approaches such as proteomics, structural proteomics and protein-protein interactions analysis. One important consideration, when moving from studying the genome and the transcriptome to the proteome, is the huge increase in potential complexity. The 4-nucleotide codes of DNA and mRNA are translated into a much more complex code of 20 amino acids, with primary sequence polypeptides of varying lengths folded into one of a startlingly large number of possible conformations and chemical modifications (e.g. phosphorylation, glycosylation and lipidation) to produce a final functional protein. Also, multiple isoforms of the same protein can be derived from alternative splicing (Figure 4).

These degrees of freedom in characterizing proteins contribute to the heterogeneity of the proteome in time and space, making the omics approach extremely challenging. In addition, techniques for protein studies are less scalable than those to study nucleic acids. In fact, in contrast with NGS and RNA-sequencing, the assessment of protein sequences cannot be currently performed at the omics scale for a number of reasons, including the following: (i) current nucleotide or protein sequence databases, used as a reference when annotating novel proteomics results, are incomplete, and sometimes inaccurate, thus irreversibly affecting the interpretation and use of newly generated data [76]; (ii) technical issues such as mass-spectrometry (MS) bias towards identification of peptides with higher concentrations, or contamination from other experiments and human keratins, and lack of uniformity across laboratories/research groups that can lead to differences in protein fragmentation and solubilization, or differences in algorithms used to run analyses [77].

### Proteomics and protein structure analysis

Proteomics is the qualitative and/or quantitative study of the proteome and is principally based on MS [78]. Proteomics is recognized for its potential to describe cell/tissue differentiation and discovery of diagnostic markers for disease; however, eventual protein function depends on how a protein is folded. 3D protein structures are generated by means of X-ray, nuclear magnetic resonance (NMR) and cryo-electron microscopy to (i) visualize protein domains, (ii) infer molecular mechanisms and protein function, (iii) study structural changes following disease-associated mutations and (iv) discover or develop drugs [79]. Researchers are encouraged to deposit data of proteomic experiments such as raw data, protein lists and associated metadata into public databases, e.g. the PRoteomics IDEntifications database [80]. A boost in the collection of proteomic data was supported by the Human Proteome Organization Proteomics Standards Initiative (HUPO-PSI, 2008) that established a communal format (mzML [81]) and a unique vocabulary for handling proteomic data [82]. As previously noted, the proteome is extremely dynamic and depends on the type of sample as well as conditions at sampling. Even when omics techniques, such as cell-wide mass spectrometry (MS), are applied, elevated sample heterogeneity complicates the comparison of different studies (e.g. overlap between transcriptome and proteome) and challenges the development of a universal and comprehensive human proteome reference. The Proteome Xchange was established as a consortium of proteomic databases to maximize the collection of proteomic experiments [83, 84]. The building of a structural proteome reference is also challenging, since methods to generate and retrieve structural data are time-consuming and low-throughput. The Protein Data Bank (PDB), with its collection of >30 000 structures for human proteins, is currently the main structural proteome repository. Of note, protein structures are dynamic, while a single conformation is a static 3D reconstruction, resulting in a partial representation of physiological and/or disease dynamics.

### Protein protein interactions

A valuable omics application is the study of protein–protein interactions (PPIs) [85, 86]. A PPI occurs when two proteins interact physically in a complex or co-localize. The growing interest in the functional prediction power of PPIs is based on the assumption that interacting proteins are likely to share common tasks or functions. PPIs are experimentally characterized, then published and catalogued in *ad hoc* repositories (e.g. PPIs databases in Pathguide). PPI databases (e.g. IntAct [87] and Biogrid [88]) are libraries where PPIs are manually annotated from peer-reviewed literature [89]. In some cases, these integrate manual curation with algorithms to predict *denovo* PPIs and text mining to automatically extract PPIs (together with functional interactions) from the literature (e.g. Search Tool for the Retrieval of Interacting Genes/Proteins [90]). HUPO-PSI (2004) started a harmonization process [91] where common formats (PSI-MI XML and MITAB) and a unique vocabulary were established to handle PPIs [92]. The International Molecular Exchange consortium (IMEx) [93] produced a unique platform (Proteomics Standard Initiative Common QUery InterfaCe) through which PPI databases within the consortium can be queried simultaneously [89, 94]. PPIs are used to build networks: within a network, each protein is defined as a node, and the connection between nodes is defined by an experimentally observed physical interaction. PPI networks provide information on the function of important protein(s) based on the guilt-by-association principle, i.e. highly interconnected proteins potentially share functional property and might be part of the same biochemical pathway(s) [95]. PPI networks can be built manually [96], allowing the merging of PPI data obtained from different sources: this approach is time-consuming, but allows the handling of the raw PPIs through custom filters and to create multi-layered networks. Some web resources (e.g. Human Integrated Protein–Protein Interaction rEference [97]) allow the generation of automated PPI networks starting from a protein or a list of proteins (i.e. seeds) selected by the user. These various platforms differ by their source of PPIs, rules for governing the merging and scoring pipelines. Finally, certain servers integrate PPIs with additional types of data including predicted interactions and co-expression data, generating hybrid networks (e.g. GeneMania [98]). Taken all together, if on one hand these multiple resources are user friendly, on the other they are not harmonized and poorly customizable leading to inconsistent results among each other. Therefore, users should thoroughly familiarize themselves with the parameters of the software, to properly extract and interpret data.

### Protein nomenclature and annotation

The comprehensive list of protein sequences, as inferred from the translation of coding regions (from nucleotide sequence repositories such as NCBI-GenBank, DNA Data Bank of Japan and EMBL-EBI) into amino acid sequences, is stored in sequence databases: some are automatically compiled [e.g. Translated EMBL Nucleotide Sequence Data Library (TrEMBL), GenBank], others are manually curated and revised (e.g. SwissProt or RefSeq). To avoid redundancy, reduce the range of different identifiers (protein IDs) and harmonize the annotation efforts, multiple databases were merged. For example, Universal Protein (UniProt [99]) acquired TrEMBL, Protein Information Resource Protein Sequence Database (PIR-PSD) and SwissProt; conversely, NCBI-nr merges annotations from GenPept, SwissProt, PIR-PSD and RefSeq.

We have summarized critical considerations in Table 4, and all web resources included in this section are shown in Supplementary Table S1c.

**Table 4.** General critical considerations on applying bioinformatics to proteomics. Problems that can be addressed by individual researchers or research groups or that should be addressed by a large community effort have been flagged with * or °, respectively.

**Observation**	**Problems**	**Proposed action**
*Protein sequences undergo revision	Changes in the gene sequence and experimental protein sequencing confirmation will result in updates to the protein sequence in protein databases Different bioinformatics tools are updated to different versions of the protein sequence databases	Always refer to the most recent protein sequence and, if old data are used, disclose the version of the protein structure of reference
°The same protein is classified through different protein IDs	Different databases classify the same protein under different IDs. This may result in mismatches between protein IDs across repositories as well as between protein and corresponding gene IDs. This causes misrepresentations or loss of relevant information	Revise the bioinformatics tools in use to allow for a comprehensive and straightforward conversion of protein IDs
°Proteins are annotated to different extents [100]	The information collected in PPI databases is derived from small-scale hypothesis-driven experiments. Therefore, there is an intrinsic bias in that less studied proteins are less reported or missing in databases (ascertainment bias)	Consider that if data for a specific protein is unavailable, this may be because such target has not been studied or annotated yet
*The proteome is dynamic [101]	Proteomic studies based on MS are normally hypothesis free but difficult to interpret, as the proteome is highly dynamic	Be aware that data might reflect a particular cellular phase, or metabolism influenced by micro-environmental stimuli
*Atlases reporting protein expression across tissues should be used carefully	Antibodies are used in immunohistochemistry to detect protein expression across different tissues. For some proteins, antibodies are not available or reliable	Consider the atlas as an indication, rely on the data only when antibodies and protocols with longer track records or those with multiple literature citations are used

## Functional annotation

Functional annotation is an analytical technique commonly applied to different types of big data (e.g. sets of genes, transcripts or proteins) to infer associated biological functions. This type of analysis, which is currently gaining notable interest and relevance, relies on the existence of manually curated libraries that annotate and classify genes and proteins on the basis of their function, as reported in the literature [102]. The most renowned and comprehensive is the Gene Ontology (GO) library that provides terms (i.e. GO terms) classified under three categories: BPs, molecular functions (MFs) and cellular components (CCs) [103]. Other libraries provide alternative types of annotation, including pathway annotation such as the Kyoto Encyclopedia of Genes and Genomes [104], Reactome [105] and Pathway Commons [106]. Conversely, regulatory annotation can be found, for example, in TRANScription FACtor [107], a library where genes are catalogued based on the transcription factors they are regulated by (the 2005 version is freely available; any subsequent version is accessible upon fee).

**Figure 5.**
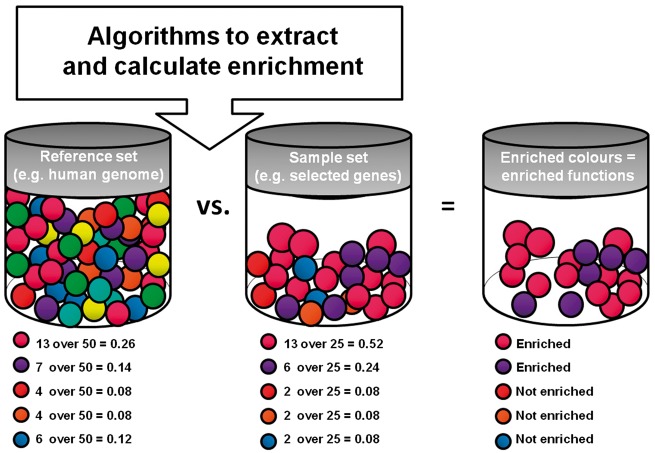
Scheme of a typical functional enrichment analysis. A sample and reference set are compared to highlight the most frequent (i.e. enriched) features within the sample set.

Functional annotation is based on a statistical assessment called enrichment. Two groups of ‘objects’ (sample versus reference set) are compared for the distribution of certain properties (e.g. functions catalogued through GO terms) [108]. If the ‘objects’ are genes, the entire genome could be used as the reference set. The latter will show a certain distribution of GO terms, reflecting the frequency of association between the catalogued BPs, MFs and CCs, and the genes in the entire genome. Conversely, the sample set is a list of genes of interest grouped together based on experimental data. The enrichment analysis compares the distribution of GO terms in the sample set (list of genes of interest) versus that observed in the reference set (genome): if a certain GO term is more frequent in the sample set than in the reference set, it is enriched, indicating functional specificity. Of note, the reference set should be tailored to the specific analysis (e.g. if assessing enrichment in the brain, the reference set should be the totality of genes known to be expressed in the brain) (Figure 5).

There is a wide variety of online portals that aid performing functional enrichment [109] (e.g. g:Profiler [110], FunRich [111], Ingenuity (accessible upon subscription fee), WebGestalt [112] and Panther [113]); each uses specific algorithms and statistical methods (e.g. Fisher’s exact or hypergeometric test corrected through Bonferroni or false discovery rate) for assessing and correcting the enrichment analysis. Each of these portals downloads groups of GO terms in its virtual space from GO and it is critical for the end user to verify the frequency at which portals perform updates. It is also important to note that any portal might be used for initial analysis; however, one should keep in mind that using the most updated portal as well as replicating analyses with a minimum of three different analytical tools is probably best practice in assessments of this kind.

We have summarized critical considerations in Table 5, and all web resources included in this section are shown in Supplementary Table S1d.

**Table 5.** General critical considerations on applying bioinformatics to functional annotation analyses. Problems that can be addressed by individual researchers or research groups or that should be addressed by a large community effort have been flagged with * or °, respectively.

**Observation**	**Problems**	**Proposed action**
*Enrichment portals run with different algorithms and statistical methods [109]	The software package chosen for the analysis (library, algorithm and statistics) will influence the final result	At the moment, there is no gold standard method for enrichment Use a minimum of three different portals to replicate and validate functional annotations
*Enrichment for GO terms may give generic results [114]	GO terms are related through family trees: general terms are umbrella terms located at the top of the tree. More specific terms are found gradually moving down towards the roots General terms are overrepresented among the results of functional enrichment	The many very general (top of the tree) GO terms might be ignored comparatively to the more specific terms (roots), as they are less likely to provide useful biological meaning(s)

## Omics beyond the central dogma and bioinformatics tools

In addition to genomics, transcriptomics and proteinomics, other areas of biomedical science are moving towards the omics scale, albeit not yet achieving the same level of complexity, depth and resolution.

### Epigenomics

There are macromolecules that bind and functionally affect the metabolism of the DNA (e.g. induction or silencing of gene expression). The Encyclopedia of DNA Elements (ENCODE) is an international endeavour with the goals of screening the entire genome and mapping every detectable functional unit [115]. This applies to coding regions, which are screened by GENCODE (a subproject of ENCODE) as well as the non-coding units. ENCODE collects results of experiments conducted to identify signature patterns, such as DNA methylation, histone modification and binding to transcription factors, suppressors and polymerases. It also collects annotation of RNA sequences that bind RBP. The final product is an atlas of DNA-based functional units, including promoters, enhancers, silencers, structural RNAs, regulatory RNAs and binding protein motives. Since signature patterns differ between cells and tissues, data are generated and collected based on cell type [116]. This valuable data set can be accessed and used directly through ENCODE or interactively through Ensembl or UCSC. Not only does ENCODE play a major role in increasing our general knowledge of the physiology and metabolism of DNA, but it also promises to provide insight into health and disease, by aiding the integration and interpretation of genomics and transcriptomics data. For example, since 88% of trait associated variants detected with GWAS fall in non-coding regions, ENCODE will tremendously impact their assessment and phenotype-related interpretation [115].

### Drugomics

Omics collections are also curated for drugs. There are databases and meta-databases (e.g. the drug–gene interaction database [117], Drug2Gene [118] and Drug Bank [119]) that collect drug–protein–gene interactions. These are useful to find existing drugs for a specific target (e.g. evaluating points of intervention within a pathway or gene/protein list), or to instantly identify all known targets of a selected drug. An additional database, part of the so-called ConnectivityMap project, provides an interface to browse a collection of genome-wide transcriptional profiles from cell cultures treated with small bioactive molecules (i.e. drugs) [120]. This resource is used as a high-throughput approach to evaluate modulation of gene expression influenced by certain drugs. This knowledge allows to identify (i) genes that are concomitantly influenced by the same drug, thus presenting with an overlapping ‘gene expression signature’ and therefore likely to share similar functions, and (ii) drugs able to influence complex biological traits of interest [121], particularly, allowing for drug repositioning [122].

### Metabolomics

Another emerging omics effort is metabolomics, the study of metabolites produced during biochemical reactions. Metabolomic databases such as the human metabolome database [123], METLIN [124] and MetaboLights [125] collect information on metabolites identified in biological samples through chromatography, NMR and MS paired with associated metadata. Of note, efforts such as the Metabolomics Standard Initiative [126] and the COordination of Standards in MetabolOmicS within the Framework Programme 7 EU Initiative [127] are currently addressing the problem of standardization of metabolomics data. Changes in the production of metabolites reflect a particular combination an individual’s genetics and environmental exposures. Therefore, they are measured in cases and controls to develop accurate diagnostics and understand relevant molecular pathways underpinning specific conditions or traits [128]. Some critical limitations apply to this field currently, including (i) the need for improvement of analytical techniques to both detect metabolites and processing results, (ii) the ongoing production of reference (and population-specific) metabolomes and (iii) the fact that we still do not completely understand the biological role of all detectable metabolites [129, 130]. Nevertheless, some promising studies have emerged: for example, profiling of lipids in plasma samples of Mexican Americans identified specific lipidic species correlated with the risk of hypertension [131]; or else, serum profiling of ovarian cancer was used to implement a support diagnostics to accurately detect early stages of the disease [132].

### Bioinformatics tools

The rise of a high number of bioinformatics tools has fostered initiatives aimed at generating portals to list them and support their effective use. For example, EBI has a bioinformatics service portal listing a variety of databases and tools tailored for specific quests or topics [133]; Bioconductor provides analysis tools and *ad hoc* scripts developed by statisticians for a variety of analyses and bioinformatics solutions; GitHUB is a free repository, easing collaboration and sharing of tools and informatics functions; OMICtools is a library of software, databases and platforms for big-data processing and analysis; Expert Protein Analysis System is a library particularly renowned for proteomics tools.

This flourishing of analytic tools and software is remarkable, and increases the speed at which data can be processed and analysed. However, with this abundance of possibilities, caution is warranted, as no single tool is comprehensive and none is infallible. It is imperative to understand the principles behind bioinformatics tools and to sensibly choose the most suitable one(s) for the purposes of the end user’s project(s).

All web resources included in this section are shown in Supplementary Table S1e.

## Perspectives

**Figure 6.**
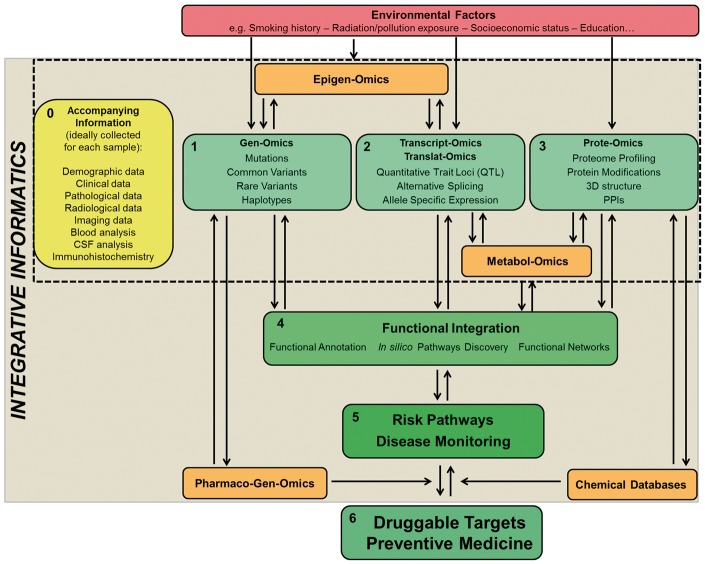
Overview on a global approach for the study of health and disease. Ideally, for individual samples, comprehensive metadata (0) should be recorded. To date, (1), (2) and (3) are being studied mainly as compartmentalized fields. A strategy to start integrating these fields currently relies on functional annotation analyses (4) that provide a valuable platform to start shedding light on disease or risk pathways (5). The influence of other elements such as epigenomics, pharmacogenomics, metabolomics and environmental factors on traits is important to have a better and more comprehensive understanding of their pathobiology. The assessment and integration of all such data will allow for the true development of successful personalized medicine (6). Color codes: green = addressed and in progress; orange = in progress; red = not yet addressed; yellow = ideal but not yet fully implemented. The gradually darker shades of green and increased font sizes indicate the expected gradual increase in the translational power of global data integration.

Advances in biomedical sciences over the past century have lent phenomenal contributions to our understanding of the human condition, providing an explanation of the causes, or even curing, a number of diseases—especially when monogenic (e.g. see Table 5 in [134]). Nevertheless, two major challenges remain unresolved in complex disorders, i.e. that of understanding their biological basis and that of developing effective treatments. Regardless of the improvements in the efficiency of data generation, the research community still struggles when stepping into the translational processes. Genomics, transcriptomics and proteinomics are still mainly separate fields that generate a monothematic type of knowledge. Nevertheless, we are witnessing the rise of inter-disciplinary data integration strategies to be applied to the study of multifactorial disorders [135]: the genome, transcriptome and proteome are, in fact, not isolated biological entities, and (multi)omics data should be concomitantly used and integrated to map risk pathways to disease (Figure 6).

### The data integration era

Integration is defined as the process through which different kinds of omics data—(multi)omics, including mutations defined through genomics, mRNA levels through transcriptomics, protein abundance and type through proteomics, and also methylation profiles through epigenomics, metabolite levels through metabolomics, metadata such as clinical outcomes, histological profiles and series of digital imaging assays and many others—are combined to create a global picture with higher informative power comparatively to the single isolated omics [136]. One of the fields at the forefront for omics data integration is cancer biology where the integrative approach is already translated to the bedside: here, implementation of data integration allowed, for example, tumour classification and subsequently prediction of aggressiveness and outcome, thus supporting the selection of personalized therapies [137]. The ColoRectal Cancer Subtyping Consortium applied data integration to a large scale, internationally collected sets of (multi)omics data (transcriptomics, genomics, methylation, microRNA and proteomics)—to classify the subtypes of colorectal cancer in biologically relevant groups—that were applied to support therapeutic decisions and predict patient outcomes [138].

Another example of integrative analysis is the production of hybrid networks combining DNA with RNA, and RNA with PPI data. In the former case, integration of DNA and RNA data has led to an improvement in matching genetic variations with their immediate effect, e.g. gene fusion or spliced isoforms [139]; in the latter, the use of transcriptome data in proteomics has increased the analytical power when transcriptome data was used to determine the mRNA content in a sample that had subsequently undergone proteome profiling and helped in accurately mapping new proteins and isoforms not reported in reference databases [140].

### Individual and collective efforts for data integration

Sometimes individual research groups set up custom pipelines to achieve data integration. For example, early attempts to couple microRNA and metabolome profiles in a tumour cell line led to the isolation of specific microRNA(s) acting as modifier(s) of cancer-associated genes [141]. Such endeavours rely on the availability of multidisciplinary experts within individual research groups and sufficient computational infrastructure supporting data storage and analysis. Having such teams allows the development of customized pipelines tailored to the specific needs; however, their efforts are not necessarily available to the wider scientific community unless shared through *ad hoc* repositories (e.g. Bioconductor and GitHUB) or in general unstructured repositories like figshare and Dryad (see Supplementary Table S1e). Emergence of scalable cloud computing platforms (Google Cloud, Amazon Web Services, Microsoft Azure) makes data storage and processing more affordable to teams that do not have sufficient in-house computing infrastructure, although such platforms require special investment.

There are also public efforts leading to the inception of a number of promising initiatives: BioSample (BioSD) is a promising tool for performing weighted harmonization among (multi)omics. Here, experiments and data sets stored within EBI databases can be queried to simultaneously access multiple types of data from the same sample, clearly representing a valuable means of simplifying data integration [142]. GeneAnalytics is a platform for querying genes against a number of curated repositories to gather knowledge about their associations with tissues, cells, diseases, pathways, GO, phenotypes, drugs and compounds [143]. This is, however, only available upon a subscription fee.

The picture is still incomplete without additional integration of other omics such as epigenomics and metabolomics: although platforms to allow integration of epigenetic with transcriptomic data (e.g. BioWardrobe [144]) are being developed, endeavours to support data optimization and sharing are welcomed. For example, the European Open Science Cloud (promoted and supported by the European Commission) represents a data repository where, through the support of expert stewards, data are standardized and stored to foster collaborative data-sharing across disciplines [145].

### Overall limitations

There are still significant biological and technical challenges impacting data integration leading to difficulties in overlapping or merging data sets and the chance to overlook potential interesting results. These limitations include the following: (i) inefficient and inconsistent nomenclatures across different databases or sources (e.g. gene or protein IDs); (ii) different data source and processing (e.g. different array or NGS platforms, differences in processing pipelines, sample preparation or study design [138]); (iii) computational power and capacity; (iv) lack of theoretical knowledge and reliable prediction models (e.g. the scarcity of models predicting metabolite changes following pathway perturbations); and (v) shortage of effective and robust pipelines to integrate and incorporate additional types of data to correct, for example, for biological differences among cell types, cell-cycle phases, tissues and developmental stages.

Also, currently, sampling material for an experiment limits the study of the biochemical life of the cell to a single snapshot exclusively accounting for the moment and condition at sampling. To start addressing issues like this, it would be ideal, in the near future, to develop tools to visualize the dynamics of CCs in 3D and include temporospatial variables that influence the behaviour of intracellular phenomena. Moreover, techniques are still in development to analyse the phenotype of cells in a high-throughput fashion, correlating changes in the genome, gene expression and the proteome to cellular phenotypes, i.e. cell-omics [146]. Another unsolved problem is that of merging data generated through different omics as it is not straightforward and requires refining steps to elaborate the data sets before integration. For example, it has been demonstrated that the transcriptome does not completely mirror the proteome of a cell [147]. Therefore, to integrate the information coming from the transcriptome and the proteome specific to a cellular phase or BP (e.g. immune response and neurotoxic response), an additional required step would be the study of the translatome, the only portion of the mRNA that is actually engaged in the translational process (i.e. interacting with the ribosomes and polysomes) isolated, for example, by ribosome profiling [148].

Finally, a major challenge to fully complete the picture is represented by environmental factors that, although recognized for critically influencing all levels of omics, still cannot be investigated through robust and reliable methods [149]. In an attempt to overcome this important issue, statisticians and epidemiologists are developing new approaches, such as Mendelian randomization through which genetic markers are used as decoys for environmental factors to be studied in association with traits or diseases [150].

### Ethics and policies in data sharing

There are still a number of issues associated with data generation and sharing, and three main levels of scrutiny apply here. First, there is a need for the donor to consent to the generation and the use of personal-level data within a study or project; also, such study/project needs to be approved by local institutional review board (IRB) Committees. After the original study is completed, sharing of data with the scientific/research community requires the initial individual consent and IRB to cover both the open data sharing and the fact that additional studies (other than the original) can be performed.

Second, raw data represent a private type of data, making it an absolute requirement to anonymize the samples through de-identification codes along with associated metadata (e.g. gender and age). Particularly, genomics (genome-wide level data), transcriptomics (in the case of raw data form which variants can be extracted) and to some extent epigenomics represent highly sensitive data since de-identification does not completely protect individual identity [151], while, in contrast, proteinomics data represents more cellular process/pathways oriented information, and if the sample is correctly anonymized there is, normally, no danger of breaching anonymity. To take genetics as an example, methods to share data might be differently regulated based on the type of data being shared: it is widely accepted to share summary statistics of a data set for further meta-analyses (where, for example, allele frequency data are not released to prevent identification of individuals); more difficult is the sharing of data sets containing individual-level raw data, as consent and approval to do so should be covered by the original IRB.

Then there is a third layer of complexity in data management that merits discussion. This regards broader ethical themes that relate to genetic counselling, including how much of the data is/can be disclosed back to the patient, and how that information is dealt within the private and professional environment. However, since the latter topic goes beyond the goals of the current review and discussion, we suggest the following reference for more details [152].

## Conclusion

In summary, there is clearly enormous potential in the integration and use of (multi)omics data for a better understanding of the molecular mechanisms, processes and pathways discriminating health and disease.

The success of this new model of science will depend on the gradual shift from a reductionist to a global approach, sustained by a lively and proactive flow of data across and between different fields of expertise, and funding programmes promoting and supporting this endeavour [153]. It is reassuring that governments are starting to acknowledge the importance of translating this comprehensive biomedical knowledge to the bedside and thus fostering the implementation of plans supporting the logistics and regulatory actions for such transformation to take place.

Together, this will eventually aid the development of measures for disease prevention, early diagnosis, disease monitoring and treatment, thus making precision medicine a forthcoming possibility.

**Key Points**

- We present an overview on the basics and exponential growth of genomics, transcriptomics and proteinomics.
- We summarize the principal bioinformatics and biostatistics tools for omics analysis.
- Genetics, functional biology, bioinformatics and biostatistics established specific jargons, impacting communication and data interpretation. We particularly aim at targeting a broad range of scientific professionals (including students) seeking knowledge outside their field of expertise.
- We provide a critical view of strengths and weaknesses of these omic approaches.

## Supplementary data

Supplementary data are available online at http://bib.oxfordjournals.org/.

## Supplementary Material

Supplementary Glossary

Supplementary Table Sup.1a-e

## Notes

**Claudia Manzoni** is a postdoc at the University of Reading whose expertise is functional biology; she has developed an interest in systems biology and bioinformatics as tool to support, guide and improve cell biology research.

## Notes

**Demis A. Kia** is a medical student undertaking a PhD at the University College London studying genetics and bioinformatics with a particular focus on Parkinson’s Disease.

## Notes

**Jana Vandrovcova** is a postdoc at the University College London with experience in genetics, transcriptomics and overall bioinformatics. Her research activities are currently focusing on the application of genetics and transcriptomics to understand pathogenesis of neurodegenerative conditions.

## Notes

**John Hardy** is professor at the University College London; his work as geneticist has been fundamental in Alzheimer’s Disease and it is now focused on the dissection of multiple neurological conditions. His contribution to genetics has been internationally recognized by the awarding of multiple prizes including the prestigious $3 million Breakthrough Prize in Life Sciences (2015).

## Notes

**Nicholas W. Wood** is Research Director of the UCL Institute of Genetics and is Galton Professor of Genetics. His primary research interest is the genetics of neurological conditions, especially Parkinson’s Disease and the Ataxias.

## Notes

**Patrick A. Lewis** is Associate Professor in Cellular and Molecular Neuroscience at the University of Reading. His research focus is the understanding of the molecular pathways underpinning Parkinson's Disease, performed through a combination of functional and systems biology.

## Notes

**Raffaele Ferrari** is a geneticist at the University College London with an interest in bioinformatics and systems biology. The main goal of his research is the dissection of the genetic underpinnings of Dementias with particular interest in Frontotemporal Dementia; he conducted the largest genome-wide association study for Frontotemporal Dementia so far and manages the International FTD-Genomics Consortium (IFGC).
