# Supplementary material for: Genome, transcriptome and proteome: the rise of omics data and their integration in biomedical sciences
Source: Brief Bioinform. 2016 Nov 22;19(2):286–302. doi: 10.1093/bib/bbw114 (PMC6018996; doi:10.1093/bib/bbw114)
Supplement: Supplementary Glossary [file bbw114_glossary_rev.docx]

| **Term** | | | | **Definition** |
| --- | --- | --- | --- | --- |
| **Build GRCh38** | | | | Current (Sept 2016) version of the human reference genome |
| **Burden analyses** | | | | A set of region-based tests used when analyzing rare variant data, where variants are grouped based on region or gene in which they are located. The information on all variants within the gene/region is then aggregated into a single score. |
| **Chip** | | | | A microarray that contains thousands of known nucleotide sequences on a glass slide, which bind to complementary sequences from a DNA or RNA sample. Provides a readout of variants in these pre-defined sequences, or quantification of RNA for expression analysis |
| **Common variants** | | | | Variations in the human genome that are common in a population. Typically used to refer to variants with a minor allele frequency > 1-5% |
| **Copy number polymorphisms** | | | | An umbrella term used to refer to any structural variation in the genome (including large insertions/deletions [indels]) |
| **Copy number variants** | | | | A form of genomic structural variation, where a segment of DNA is deleted or multiplicated, resulting in variation in the number of copies of that segment in the genome (small indels) |
| **Coverage** | | | | Number of times a nucleotide or a stretch of bases is read during the sequencing process |
| **Enrichment** | | | | Statistical process to evaluate if a term is more frequently present (enriched) in a sample set when compared with a reference set |
| **Entrez Gene IDs** | | | | Code used to identify nucleotide sequences as they are collected in the NCBI Gene database |
| **Epigenome** | | | | Total set of DNA modifications (e.g. DNA methylation and histone acetylation) of an organism/individual |
| **Exon** | | | | A transcribed region of a gene that is present in mature messenger RNA |
| **Expression-based quantitative trait loci (eQTL)** | | | | Method to assess the expression level of a gene is controlled/influenced by a polymorphic region of the DNA |
| **Functional annotation** | | | | Evaluation of biological functions associated with a set of genes or transcripts or proteins |
| **Genetic risk scoring** | | | | Method to generate a risk score for an individual with respect to an outcome/disease based on their genotype: in its simplest form, it is the simultaneous assessment of multiple genes/alleles (i.e., a gene signature) associated with the phenotype/trait/condition. |
| **Genome** | | | | Total set of DNA elements of an organism/individual |
| **GWAS** | | | | Genome Wide Association Study is a method to assess whether common variations in the population are associated with a specific phenotypic trait |
| **Haplotype** | A set of genetic variants that tend to be inherited together | | | |
| **Heritability** | The proportion of phenotypic variance explained by genetic variance | | | |
| **Imputation** | Inference of untyped genetic variants in an individual using surrounding known genotypes and information about haplotypes in the population | | | |
| **Interactome** | Total set of proteins able to physically interact with a protein of interest (seed of the interactome) | | | |
| **Intron** | A non-transcribed region of a gene that is spliced out from the mature messenger RNA. | | | |
| **Linkage disequilibrium (LD)** | The occurrence of specific combinations of alleles at two or more linked loci more frequently than expected by chance from the frequency of the allele in the population quantified as r^2^ or D’ | | | |
| **Mass-spectrometry** | Biophysical technique through which proteins are recognized by their mass or the mass of their fragments | | | |
| **Metadata** | Collection of information regarding a sample under analysis | | | |
| **Minor allele frequency (MAF)** | The frequency of the least common allele of a polymorphism in a given population | | | |
| **Microarray Vs Sequencing** | Microarray is any technique based on a set of pre-defined probes to which the sample is tested against. Its counterpart is the sequencing technique where the molecules in the sample are sequenced base by base without depending on prior knowledge | | | |
| **Network** | Mathematical object containing nodes connected by edges. A connection between 2 nodes indicates a specific relation/interaction between them | | | |
| **Next-generation sequencing (NGS)** | Sequencing technique. Fragments from the entire genome are sequenced base by base and then re-united following indications in a reference genome | | | |
| **Node** | Object composing a network | | | |
| **Oligonucleotide** | A short DNA or RNA fragment made of fewer than 30 bases | | | |
| **Ontology** | A major bioinformatics initiative to annotate and harmonize the relation between gene(s) and gene product(s) attributes across all species | | | |
| **Penetrance** | The proportion of individuals with a mutation who also exhibit the phenotype associated with the mutation | | | |
| **Pharmacogenomics** | The study of the influence of the genetic architecture on drug efficacy | | | |
| **Primary protein structure** | Amino acid sequence | | | |
| **Probe** | Short fragment of DNA or RNA. Its nucleobase sequence is used to hybridize with a target fragment of DNA or RNA | | | |
| **Protein Primary Structure** | Ordered sequence of the amino acids composing a given protein | | | |
| **Proteinomics** | The study of the proteome, investigating features such as composition (type/amount of proteins), chemical modifications, 3D structure and protein interactions | | | |
| **Proteome** | Total set of proteins (type and amount) in a cell/tissue/biological sample | | | |
| **Proteomics** | Analysis, normally through mass spectrometry, of the total set of proteins (type and amount) in a cell/tissue/biological sample | | | |
| **Rare variants** | Variations in the human genome that are rare in a population. Typically used to refer to variants with a minor allele frequency < 1% | | | |
| **Regression model** | | A statistical process that aims to find the mathematical relationship between an independent variable (or predictor) with a dependent variable (or outcome) | | |
| **Sanger sequencing** | | A DNA sequencing method, where the DNA to be sequenced is used as a template for DNA synthesis. Chain terminating dideoxynucleotides are included in the reaction, leaving DNA fragments of different sizes, each ending with a fluorescently labelled known nucleotide. These fragments can then be ordered by size and the DNA sequence read by laser on the basis of the labelled nucleotides | | |
| **Secondary protein structure** | | The amino acid, primary sequence is twisted in 3D by the formation of hydrogen bonds among distant amino acid residues | | |
| **Seed** | | Node used as starting point for an analysis | | |
| **Simple nucleotide variations** | | A term sometimes used to refer to SNPs and small insertions/deletions (indels) | | |
| **Single nucleotide polymorphisms (SNPs)** | | Single change of one of the DNA bases. Point mutation in a DNA sequence | | |
| **Small insertion/deletions** | | Insertion/deletion of a stretch of few bases in a sequence of DNA | | |
| **Splicing-based quantitative trait loci (sQTL)** | | Method to assess the splicing of an m-RNA controlled/influenced by a polymorphic region of the DNA | | |
| **Structural variations** | | Variations affecting a larger region in the DNA sequence (typically more than 1 kb), including deletions, duplications, insertions, inversions, and translocations | | |
| **Tertiary protein structure** | | The secondary structure of a protein is further twisted and different portions (domains) assume reciprocal position in the space | | |
| **Transcriptome** | | Total set of ribonucleic-acid (RNA) transcripts in a cell/tissue | | |
| **Weighted gene co-expression network analysis (WGCNA)** | | Network composed of genes (nodes) as found co- expressed (connected) in a certain sample | | |
| **Whole exome sequencing (WES)** | | A type of NGS in which exons are sequenced | | |
| **Whole genome sequencing (WGS)** | | A type of NGS in which the entire genome is sequenced | | |
