# Supplementary material for: Genome, transcriptome and proteome: the rise of omics data and their integration in biomedical sciences
Source: Brief Bioinform. 2016 Nov 22;19(2):286–302. doi: 10.1093/bib/bbw114 (PMC6018996; doi:10.1093/bib/bbw114)
Supplement: Supplementary Table Sup.1a-e [file bbw114_table_sup.1a-e_rew.docx]

**Supplementary Table 1a**

| Resource | | Description | | Repository | Link | Access |
| --- | --- | --- | --- | --- | --- | --- |
|  | | | **Organizations** | | |  |
| EMBL | European Molecular Biology Laboratory | | | YES | http://www.embl.org/ | Free |
| NCBI | National Center for Biotechnology Information (NIH based - USA) | | | YES | http://www.ncbi.nlm.nih.gov/ | Free |
| Genome Browsers | | | | | | |
| Ensembl | Eukaryotic Genome databases (EMBL based) | | | NO | http://www.ensembl.org/index.html | Free |
| UCSC | UCSC Genome Browser, database of reference sequences (University of California Santa Cruz) | | | NO | https://genome.ucsc.edu/ | Free |
| NCBI-Genome | National Center for Biotechnology Information (NIH based - USA) | | | NO | http://www.ncbi.nlm.nih.gov/genome/ | Free |
|  | | | **Nucleotide Sequences and Reference Genomes** | | |  |
| DDBJ | DNA Data Bank of Japan | | | YES | http://www.ddbj.nig.ac.jp/ | Free |
| EMBL-EBI | European Nucleotide Archive, provides records of the world's nucleotide sequencing information (EMBL based) | | | YES | http://www.ebi.ac.uk/ena | Free |
| GenBank | Annotated collection of all publicly available DNA sequences (NCBI based) | | | YES | http://www.ncbi.nlm.nih.gov/genbank/ | Free |
| RefSeq | Comprehensive, integrated, non-redundant, well-annotated set of sequences. RefSeq genomes are copies of selected assembled genomes in GenBank (NCBI based) | | | NO | http://www.ncbi.nlm.nih.gov/refseq/ | Free |
| HGP | Human Genome Project | | | NO | <http://www.genome.gov/11006929> | Free |
| HapMap | Haplotype Map Project | | | NO | <http://hapmap.ncbi.nlm.nih.gov/> | Free |
| 1000 Genomes | 1000 Genomes Project | | | NO | <http://www.1000genomes.org/> | Free |
| UK10K | The study of the exome of 10,000 people with selected extreme phenotypes (UK) | | | NO | http://www.uk10k.org/ | Permission required |
| 100,000 Genomes | The study of the whole genome of >70,000 people with rare diseases and cancer to benefit genomic medicine and research (UK) | | | NO | http://www.genomicsengland.co.uk/ | Permission required |
| Precision Medicine Initiative | Precision medicine initiative (US) involving the health records and whole genome sequence of one million volunteers to fight cancer and rare diseases (US) | | | NO | https://www.nih.gov/precision-medicine-initiative-cohort-program | To be established |
|  | | | **Other Genomic Data** | | |  |
| dbGaP | Archive for the distribution of genomics data (NCBI based) | | | YES | http://www.ncbi.nlm.nih.gov/gap | Free |
| dbSNP | Catalogue of SNP (NCBI based) | | | YES | http://www.ncbi.nlm.nih.gov/SNP/ | Free |
| ClinVar | Archive for genomic variation in human health (NCBI based) | | | YES | http://www.ncbi.nlm.nih.gov/clinvar/ | Free |
| EGA | European Genome-phenome Archive. To archive, process and distribute genetic data (EMBL based) | | | YES | https://www.ebi.ac.uk/ega/submission | Free |
| EVA | European Variation Archve (EMBL based) | | | YES | http://www.ebi.ac.uk/eva/ | Free |
| ExAC | Collection of exome sequencing data from large-scale sequencing projects | | | NO | http://exac.broadinstitute.org/ | Free |
| GWAS catalogue | Quality controlled, manually curated, collection of published genome-wide association studies (EMBL based) | | | NO | https://www.ebi.ac.uk/gwas/ | Free |
| OMIM | Human genes to genetic phenotypes | | | NO | http://www.omim.org/about | Free |
|  | | | **Platform to access Bioinformatics Resources and Tools** | | |  |
| Sanger Imputation Service | Free genotype imputation and phasing service provided by the Wellcome Trust. It provides access to the latest haplotypes from the Haplotype Reference Consortium | | | NO | [https://imputation.sanger.ac.uk](https://imputation.sanger.ac.uk/) | Free |
| Michigan Imputation Service | Free genotype imputation and phasing service. It provides access to the latest haplotypes from the Haplotype Reference Consortium | | | NO | https://imputationserver.sph.umich.edu/index.html | Free |
|  |  | | |  |  |  |
| Bioconductor | Resource for using or sharing working packages and/or pipelines | | | YES | https://www.bioconductor.org/ | Free |
| GATK | Software for analysis of high-throughput sequencing data | | | NO | https://www.broadinstitute.org/gatk/ | Free |
| Plink | whole genome association analysis toolset | | | NO | http://pngu.mgh.harvard.edu/~purcell/plink/ | Free |
| Snptest | Analysis of single SNP association in GWAS | | | NO | https://mathgen.stats.ox.ac.uk/genetics_software/snptest/snptest.html | Free |

**Supplementary Table 1b**

| Resource | | Description | | Repository | Link | Access |
| --- | --- | --- | --- | --- | --- | --- |
|  | | | **Organizations** | | |  |
| EMBL | European Molecular Biology Laboratory | | | YES | http://www.embl.org/ | Free |
| NCBI | National Center for Biotechnology Information (NIH based - USA) | | | YES | http://www.ncbi.nlm.nih.gov/ | Free |
|  | | | **Platform to access Bioinformatics Resources and Tools** | | |  |
| Bioconductor | Resource for using or sharing working packages and/or pipelines | | | YES | https://www.bioconductor.org/ | Free |
|  | | | **Providers of RNA Sequencing/Microarrays Platforms** | | |  |
| Affymetrix | Affymetrix® microarray solutions | | | NO | http://www.affymetrix.com/estore/ | £ |
| Agilent | Microarrays and sequencing | | | NO | http://www.agilent.co.uk/home | £ |
| Illumina | Microarrays and sequencing | | | NO | http://www.illumina.com/ | £ |
| ION Torrent | Thermo Fisher Scientific, sequencing | | | NO | https://www.thermofisher.com/uk/en/home/brands/ion-torrent.html | £ |
|  | | | **Repositories of Transcriptome Data** | | |  |
| Braineac | Catalog of human gene expression in the brain | | | NO | http://www.braineac.org/ | Free |
| GEO | Archive of array- and sequence-based expression data (NCBI based) | | | YES | http://www.ncbi.nlm.nih.gov/geo/ | Free |
| GTEx | Catalog of human gene expression | | | NO | http://www.gtexportal.org/home/ | Free |
| EBI | ArrayExpress | | | YES | https://www.ebi.ac.uk/arrayexpress/ | Free |
|  | Expression Atlas | | | NO | https://www.ebi.ac.uk/gxa/home | Free |
| Ensembl | Eukaryotic Genome databases (EMBL based) | | | YES | http://www.ensembl.org/info/website/tutorials/expression.html | Free |
| HBA | Human Brain Atlas, tissue-specific expression patterns of genes of interest at different stages of life | | | NO | http://hbatlas.org/ | Free |
| MicroRNA and mRNA Integrative Analyses | | | | | | |
| MAGIA | web tool for mirna-genes integrated analysis | | | NO | http://gencomp.bio.unipd.it/magia2/start/ | Free |
| GenMiR++ | Bayesian statistical methodfor microRNA targets | | | NO | http://www.psi.toronto.edu/genmir/ | Free |
| mirConnX | interface for inferring, displaying and parsing mRNA and microRNA (miRNA) gene regulatory networks | | | NO | http://www.benoslab.pitt.edu/mirconnx/ | Free |

**Supplementary Table 1c**

| Resource | | Description | | Repository | Link | Access |
| --- | --- | --- | --- | --- | --- | --- |
|  | | | **Databases of Protein Sequences** | | |  |
| PIR-PSD | Protein Information Resource – Protein Sequence Database (revised) annotations have been integrated into UniProt | | | NO | http://pir.georgetown.edu/pirwww/dbinfo/pir_psd.shtml | Free |
| RefSeq | Comprehensive, integrated, non-redundant, well-annotated set of sequences, including genomic DNA, transcripts, and proteins. Protein records are generated by computation, manual curation, propagation from annotated genomes. (NCBI based) | | | NO | http://www.ncbi.nlm.nih.gov/refseq/about/ | Free |
| SwissProt | Manually curated and annotated protein records in UniProt. | | | NO | http://www.uniprot.org/uniprot/ | Free |
| TrEMBL | Automatically annotated (un-revised) protein records in UniProt | | | NO | http://www.uniprot.org/uniprot/ | Free |
| UniProt | Universal Protein Repository with protein sequence and functional information Contains non-redundant protein sequences from different databases | | | NO | http://www.uniprot.org/ | Free |
|  | | | **Repositories of Proteomics Studies** | | |  |
| PRIDE | Proteomics Identifications database for proteomics data and post-translational modifications | | | YES | http://www.ebi.ac.uk/pride/archive/ | Free |
| PX | Proteome Xchange Consortium | | | YES | http://www.proteomexchange.org/ | Free |
|  | | | **3D Protein Structures** | | |  |
| PDB | Protein Data Bank – repository of 3D protein structures | | | YES | http://www.rcsb.org/pdb/home/home.do | Free |
| RasMol | Application tool to visualize crystal structure within PDB | | | NO | http://www.openrasmol.org/ | Free |
|  | | | **PPIs Databases** | | |  |
| Biogrid | PPIs database | | | NO | http://thebiogrid.org/ | Free |
| IMEx | International Molecular Exchange consortium of PPIs resources | | | NO | http://www.imexconsortium.org/ | Free |
| IntAct | PPIs database | | | NO | http://www.ebi.ac.uk/intact/ | Free |
| Mentha | Meta-database for PPIs | | | NO | http://mentha.uniroma2.it/about.php | Free |
| Pathguide | Biological pathway and molecular interaction related resources | | | NO | http://www.pathguide.org/ | Free |
| PSICQUIC | Unique query platform for members of the IMEx consortium | | | NO | http://www.ebi.ac.uk/Tools/webservices/psicquic/view/main.xhtml | Free |
| STRING | Database of known and predicted protein interactions and functional associations | | | NO | http://string-db.org/ | Free |
| PPI Networks Construction | | | | | | |
| HIPPIE | Online platform for PPIs network construction | | | NO | http://cbdm-01.zdv.uni-mainz.de/~mschaefer/hippie/ | Free |
| GeneMania | Online platform for functional network construction | | | NO | http://www.genemania.org/ | Free |

**Supplementary Table 1d**

| Resource | | Description | | Repository | Link | Access |
| --- | --- | --- | --- | --- | --- | --- |
|  | | | **Ontologies of Gene Product Functions** | | |  |
| Gene Ontology | Library for description of gene products for their associated biological processes (BP), cellular components (CC) and molecular functions (MF) | | | NO | http://geneontology.org/ | Free |
|  | | | **Databases of Pathways** | | |  |
| KEGG | Kyoto Encyclopedia of Genes and Genomes, pathways database | | | NO | http://www.genome.jp/kegg/ | Free |
| Reactome | Curated pathways database | | | NO | http://www.reactome.org/ | Free |
| Pathway Commons | Single access point to query biological pathway databases | | | NO | http://www.pathwaycommons.org/about/ | Free |
| Repository of Transcription Factors | | | | | | |
| TRANSFAC | Database of eukaryotic transcription factors | | | NO | http://www.gene-regulation.com/pub/databases.html | £ |
|  | | | **Platform for Enrichment** | | |  |
| g:Profiler | Bioinformatics platform to functionally analyze gene/transcript/protein lists; (web-based enrichment analysis tool) | | | NO | http://biit.cs.ut.ee/gprofiler/ | Free |
| Ingenuity | Commercial application to functionally analyze gene/transcript/protein lists; (web-based enrichment analysis tool) | | | NO | Ingenuity | £ |
| WebGestalt | Bioinformatics platform to functionally analyze gene/transcript/protein lists; (web-based enrichment analysis tool) | | | NO | http://bioinfo.vanderbilt.edu/webgestalt/ | Free |
| FunRich | Bioinformatics platform to functionally analyze gene/transcript/protein lists; (web-based enrichment analysis tool) | | | NO | http://www.funrich.org/ | Free |
| Panther | Bioinformatics platform to functionally analyze gene/transcript/protein lists; (web-based enrichment analysis tool) | | | NO | http://pantherdb.org/ | Free |

**Supplementary Table 1e**

| Resource | | Description | | Repository | Link | Access |
| --- | --- | --- | --- | --- | --- | --- |
|  | | | **Epigenomics** | | |  |
| ENCODE | List of functional elements in the human genome | | | YES | https://genome.ucsc.edu/ENCODE/ | Free |
|  | | | **Drugs** | | |  |
| ChEMBL | ChEMBL is a database of bioactive drug-like small molecules | | | NO | https://www.ebi.ac.uk/chembl/ | Free |
| DGIdb | Interface for searching lists of genes against the known or potential drug-gene interactions | | | NO | http://dgidb.genome.wustl.edu/ | Free |
| DrugBank | Detailed drug-target archive | | | NO | http://www.drugbank.ca/ | Free |
| Drug2Gene | Relations between genes/proteins and drugs/compounds | | | NO | http://drug2gene.info/ | Free |
| ConnectivityMap | Genome-wide transcriptional profiles from cell cultures treated with small bioactive molecules | | | NO | http://portals.broadinstitute.org/cmap/ | Free |
| Metabolomics | | | | | | |
| HMDB | Database of small molecule metabolites in the human body | | | NO | http://www.hmdb.ca/ | Free |
| METLIN | Repository of metabolite information and tandem mass spectrometry data | | | NO | https://metlin.scripps.edu/index.php | Free |
| MetaboLights | a database for Metabolomics experiments and derived information | | | YES | http://www.ebi.ac.uk/metabolights/ | Free |
|  | | | **Resources and Tools** | | |  |
| Bioconductor | | Resource for using or sharing working packages and/or pipelines | | YES | https://www.bioconductor.org/ | Free |
| EMBL-EBI | | List of bioinformatics services and tools supported by EBI | | NO | http://www.ebi.ac.uk/services | Free |
| ExPASy | | Bioinformatics resource portal to access bioinformatics tools for –omics analysis, curated by the Swiss Institute of Bioinformatics | | NO | http://www.expasy.org | Free |
| GitHUB | | Large collection of open source software | | YES | https://github.com/ | Free |
| OMICtools | | Platform for accessing bioinformatics tools for –omics analysis | | YES | http://www.omicstool.com | Free |
| Pathguide | | Platform for accessing biological pathway and molecular interaction related resources | | YES | http://www.pathguide.org/ | Free |
| figshare | | Cloud solution for data storage and sharing | | YES | https://figshare.com/ | £ |
| Dryad | | Cloud solution for data storage and sharing | | YES | http://datadryad.org/ | £ |
